# Risk factors for rescue analgesic use on the first postoperative day after upper limb surgery performed under single-injection brachial plexus block: a retrospective study of 930 cases

**DOI:** 10.1186/s40981-017-0110-9

**Published:** 2017-07-27

**Authors:** Tatsunori Watanabe, Koji Moriya, Takuya Yoda, Naoto Tsubokawa, Andrey B. Petrenko, Hiroshi Baba

**Affiliations:** 1 Division of Anesthesiology, Niigata University Graduate School of Medical and Dental Sciences, 1-754 Asahimachi-dori, Chuo-ku, Niigata, 951-8520 Japan; 2 Niigata Hand Surgery Foundation, Seiro, Japan; 3 Department of Orthopedics, Uonuma Institute of Community Medicine, Niigata University Medical and Dental Hospital, Minami-Uonuma, Japan

**Keywords:** Upper limb surgery, Brachial plexus block, Rescue analgesia, Risk factors, Logistic regression model

## Abstract

**Background:**

Postoperative pain management after upper limb surgery is important for preventing adverse events that can prolong hospital stay and cause readmission. This study aimed to identify the risk factors associated with rescue analgesic use on the first postoperative day after upper limb surgery performed under single-injection brachial plexus block (BPB).

**Findings:**

We retrospectively analyzed records from 930 patients who underwent upper limb surgery under a single-injection BPB. Postoperatively, patients were administered oral loxoprofen regularly and rescue analgesics when analgesia was insufficient. We assessed the association between patient, surgical information, and rescue analgesic use on the first day after surgery (from 7:00 PM on the day of surgery to 7:00 AM on the first postoperative day), using a logistic regression model. Multivariate analysis revealed a significant association between rescue analgesic use and bone surgery, in particular, osteotomy, ligament repair and reconstruction, osteosynthesis, treatment for an amputated digit, and surgical duration.

**Conclusion:**

Bone surgery (osteotomy, ligament repair and reconstruction, osteosynthesis, treatment for an amputated digit) and a longer operative time were risk factors for rescue analgesic use for treating postoperative pain after upper limb surgery performed under single-injection BPB.

## Findings

### Introduction

Postoperative pain management after upper limb surgery is important to prevent adverse events such as delayed postoperative function, thereby reducing the duration of hospital stays and minimizing the occurrence of readmission. In upper limb surgery, brachial plexus blocks (BPBs) are often performed for anesthesia and postoperative analgesia. A single injection of BPB with long-acting local anesthetics and regular administration of nonsteroidal anti-inflammatory drugs (NSAIDs) is effective at reducing postoperative pain [1]. In Japan, an oral formulation of loxoprofen, a NSAID, is often used to treat postoperative pain [2, 3] because patients who undergo surgery under BPB have no restrictions on food intake and oral loxoprofen causes significantly less nausea compared to opioid analgesics. However, patients with more intense postoperative pain despite receiving loxoprofen may require rescue analgesics.

The risk factors for postoperative pain have been analyzed in previous studies, and it is generally accepted that the degree of postoperative pain depends on various parameters, such as age, sex [4, 5], surgical procedure, and surgical site [6]. However, these studies used broad classifications based on the following: the type of surgery according to anatomical regions, surgical discipline, or scale of surgery (minor or major). The specific risk factors for the use of rescue analgesics to treat postoperative pain in upper limb surgery are unknown. In the upper limb, there are various components, such as the muscles, nerves, and blood vessels, and surgical sites vary and include the hand, wrist, elbow, etc. Moreover, in upper limb surgery, the age of the patients varies significantly.

Therefore, we set out to investigate the risk factors for rescue analgesic use in upper limb surgery. To this end, we retrospectively analyzed, using a logistic regression model, the association between patient and surgical data and additional analgesic administration on the first day after upper limb surgery performed under single-injection BPB.

## Materials and methods

### Data collection

Data were obtained from medical records retrospectively. Data were collected on the use of rescue analgesics during the first postoperative night (from 7:00 PM on the day of surgery to 7:00 AM on the first postoperative day, based on the established practice of regular oral administration of loxoprofen at supper and in the morning at our facility). Additionally, data on patient age, sex, surgical procedure, surgical site, duration of surgery, and duration of tourniquet placement were collected. Moreover, information on the use of additional local anesthetics during surgery was collected because such cases were suspected to be BPB failures.

### Subjects

**Fig. 1:**
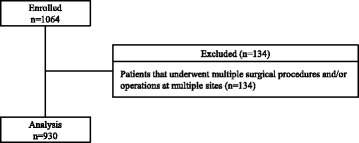
A flow chart showing the process of patient enrollment and subsequent analysis

A total of 1064 adult patients who were hospitalized, surgically treated under a single-injection BPB, and administered oral loxoprofen regularly three times a day starting at supper on the day of surgery between July 2012 and March 2014 were included. Patients who underwent multiple surgical procedures and/or operations at multiple sites (*n* = 134) were excluded; thus, 930 patients were analyzed (Fig. 1). Although most patients underwent surgical procedures that are typically performed on an outpatient basis, they were still hospitalized after the surgery, which is common in Japan owing to national medical insurance coverage.

### Perioperative anesthesia and analgesia

**Fig. 2:**
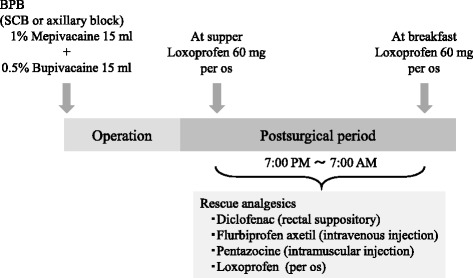
Pre- and postoperative analgesic regimen. *BPB* brachial plexus block, *SCB* supraclavicular block, *7:00 PM~7:00 AM* from 7:00 PM on the day of surgery to 7:00 AM on the first postoperative day

All blocks were performed prior to surgery. A single-injection BPB was performed under landmark or ultrasound guidance by a supraclavicular and axillary approach with 1% mepivacaine (15 mL) and 0.5% bupivacaine (15 mL; total volume, 30 mL). If the patient experienced pain in the surgical field during surgery, local infiltration or finger or wrist blocks were added using 10 mL of 0.5 or 1% lidocaine or 1% mepivacaine. Postoperatively, regular administration of oral loxoprofen, three times per day, was started at supper. When patients complained of pain, the following rescue analgesics were administered according to the patients’ preferences: a diclofenac suppository, intravenous flurbiprofen axetil, intramuscular pentazocine, or oral loxoprofen (Fig. 2).

### Classification of the surgical procedure

Surgical procedures were first classified into broad and subsequently, into narrow subgroups.

The procedures were broadly classified into either bone surgery-related procedures, used mostly for bones and joints, or soft tissue surgery-related procedures, which did not involve bone manipulation. Bone surgeries were further categorized as osteotomy (a surgical procedure of cutting and fixing the bone), osteosynthesis (surgery after fracture with or without arthroscopic assistance), arthroplasty and mobilization (a surgical procedure to restore the range of joint motion by excising a bony spur), arthrodesis (surgical joint fusion to relieve pain without osteotomy), ligament repair and reconstruction (surgery using a bone suture anchor with or without arthroscopic assistance), arthroscopic surgery, treatment for an amputated digit, implant removal to fix bones or joints, or other bone surgeries (other procedures such as the removal of a free body). All categories of surgical procedures are shown in Table 1.

**Table 1:** Classification of surgical procedures

Broad classification	Narrow classification	Surgical procedure
Bone surgery	Osteotomy	Sauve-Kapandji procedure Corrective osteotomy Shortening osteotomy
	Osteosynthesis	Open reduction and internal fixation (ORIF)
	Arthroplasty and mobilization	Arthroplasty (MP, CM wrist joint) Mobilization (MP joint)
	Arthrodesis	Arthrodesis (CM, PIP, DIP, IP joint)
	Ligament repair and reconstruction	Repair or reconstruction of TFCC Repair or reconstruction of collateral ligament (Elbow, PIP, and MP joint) Open reduction of dislocation joint
	Arthroscopic surgery	Examination Synovectomy
	Treatment for amputated digit	Stump plasty Local artery flap combined with bone excision
	Removal of implants to fix bones or joints	Removal of implants to fix bone or joint
	Other bone surgery	Limited osteotomy Excision of osteophytes Curettage of bone tumor Removal of free body Osteomyelitis debridement Synovectomy (PIP, IP joint) Joint mobilization surgery (MP, PIP, Wrist, Elbow joint) Arthrorisis
Soft tissue surgery	Soft tissue surgery	Neurolysis Neurorrhaphy Removal of subcutaneous foreign body Debridement Excision of ganglion Dupuytren’s contracture surgery Excision of soft tissue tumor Web plasty Opponensplasty Tendon sheath reconstruction Tenolysis Carpal tunnel release Z-plasty Nerve transfer Thenar flap Tendon transfer Tendon repair Severance of flap Venous flap Venous wrapping of repaired nerve Arterial anastomosis Defatting Wound treatment Cross finger flap Synovectomy of tendon Pulp plasty Hemangioma resection Tendon graft Skin graft Finger stem dissection

*MP* metacarpal phalangeal, *CM* carpometacarpal, *PIP* proximal interphalangeal, *DIP* distal interphalangeal, *IP* interphalangeal, *TFCC* triangular fibrocartilage complex

### Classification of the surgical site

The surgical site was classified as the hand, wrist, forearm, elbow, or upper arm. The hand was defined as being distal to the carpus. The wrist was defined as extending from the carpus to the distal one-third of the forearm. The forearm was defined as the middle forearm, and the elbow was defined as extending from the proximal one-third of the forearm to the distal one-third of the upper arm. The upper arm was defined as the proximal two-thirds of the upper arm.

### Data analysis

To determine the risk factors for rescue analgesic administration, we used single- and multiple-explanatory-variable logistic regression models. Variables that were significantly associated with rescue analgesia use in a single-explanatory-variable logistic regression model were entered and further analyzed in the multi-explanatory-variable logistic regression model. Single- and multiple-explanatory-variable logistic regression models were obtained with analgesic use as the outcome variable (1, used; 0, not used). The patients’ age, duration of surgical procedure, and tourniquet placement were analyzed as continuous variables. Sex, additional local anesthetic use during surgery, and the broadly classified surgical procedures were analyzed as single-indicator variables (1 or 0). The surgical site and narrowly classified surgical procedures were analyzed as categorical variables. Variables with *P* values less than 0.05 in univariate analysis were then evaluated in a multivariate logistic regression model. Odds ratios (OR), 95% confidence intervals (CI), and *P* values were calculated. *P* values less than 0.05 were considered significant.

## Results

### Patient characteristics

The average age of the 930 patients (537 men and 393 women) was 54 ± 16 years (mean ± standard deviation).

### Logistic regression analysis

Rescue analgesics were administered in 148 patients (15.9%). First, the single-explanatory-variable logistic regression model (Table 2) indicated a significant association between rescue analgesic use on the first day after surgery and female sex, duration of surgical procedure, duration of tourniquet placement, wrist surgery as the surgical site category, bone surgery in the broad classification of surgical procedures, and a few procedures in the narrow classification, namely osteotomy, ligament repair and reconstruction, osteosynthesis, treatment for an amputated digit, and other bone surgery. No significant association was observed between rescue analgesic use and age and additional local anesthetic use during surgery.

**Table 2:** Single explanatory variable logistic regression models

	*n*	OR	95% CI	
Patient background				
Female (vs. male)		1.82	1.28–2.59	*P* < 0.05
Age (per 10 years old increase)		1.02	0.92–1.13	*P* = 0.77
Additional local anesthetic use during surgery (vs. disuse)		1.68	0.88–2.96	*P* = 0.12
Duration				
Surgical duration (per 30 min increase)		1.56	1.32–1.85	*P* < 0.05
Tourniquet duration (per 30 min increase)		1.89	1.56–2.29	*P* < 0.05
Surgical site				
Hand	429	1.00	Ref	–
Wrist	377	2.28	1.55–3.35	*P* < 0.05
Forearm	30	0.57	0.13–2.46	*P* = 0.45
Elbow	91	1.44	0.76–2.75	*P* = 0.26
Upper arm	3	n.c	–	–
Broad classification				
Bone surgery	580	4.14	2.57–6.65	*P* < 0.05
Soft tissue surgery	350	1.00	Ref	–
Narrow classification				
Osteotomy	26	9.32	3.79–22.9	*P* < 0.05
Ligament repair and reconstruction	28	8.28	3.42–20.1	*P* < 0.05
Osteosynthesis	248	6.84	4.12–11.4	*P* < 0.05
Treatment for amputated digit	42	4.07	1.73–9.55	*P* < 0.05
Arthrodesis	20	3.73	1.15–12.1	*P* < 0.05
Arthroplasty	6	2.98	0.33–26.6	*P* = 0.33
Other bone surgery	31	2.87	1.00–8.19	*P* < 0.05
Arthroscopic surgery	15	1.07	0.13–8.48	*P* = 0.95
Soft tissue surgery	350	1.00	Ref	–
Removal of implants to fix bone or joint	164	0.77	0.33–1.76	*P* = 0.53

*Ref* reference, *OR* odds ratio, *CI* confidence interval

Next, multiple-explanatory-variable logistic regression analysis was performed. Statistically significant variables from the single-explanatory-variable logistic regression analysis were entered into and further analyzed by the multi-explanatory-variable logistic regression models. Duration of tourniquet placement and broadly classified surgical procedures were not included in this analysis to avoid collinearity. No significant association was observed between rescue analgesic use and female sex or wrist surgery in the surgical site category and other bone surgery category in the narrow classification of surgical procedures (Table 3). However, a significant association was observed between rescue analgesic use and duration of surgical procedure and a few procedures in the narrow classification of surgical procedures, osteotomy, ligament repair and reconstruction, osteosynthesis, and treatment for an amputated digit.

**Table 3:** Multiple explanatory variable logistic regression models

	*n*	OR	95% CI	
Patient background				
Female (vs. Male)		1.44	0.94–2.21	*P* = 0.10
Duration				
Surgical duration (per 30 min increase)		1.40	1.13–1.73	*P* < 0.05
Surgical site				
Hand	429	1.00	Ref	–
Wrist	377	1.61	0.95–2.71	*P* = 0.08
Forearm	30	0.63	0.14–2.91	*P* = 0.55
Elbow	91	1.58	0.77–3.26	*P* = 0.21
Upper arm	3	n.c	–	–
Narrow classification				
Osteotomy	26	6.07	2.30–16.1	*P* < 0.05
Ligament repair and reconstruction	28	6.99	2.79–17.5	*P* < 0.05
Osteosynthesis	248	5.18	2.97–9.01	*P* < 0.05
Treatment for amputated digit	42	5.94	2.43–14.5	*P* < 0.05
Arthrodesis	20	2.80	0.83–9.40	*P* = 0.10
Arthroplasty and mobilization	6	1.61	0.17–15.5	*P* = 0.68
Other bone surgery	31	2.77	0.95–8.04	*P* = 0.06
Arthroscopic surgery	15	1.02	0.12–8.44	*P* = 0.99
Soft tissue surgery	350	1.00	Ref	–
Removal of implants to fix bone or joint	164	0.69	0.28–1.73	*P* = 0.43

*Ref* reference, *OR* odds ratio, *CI* confidence interval

## Discussion

This study demonstrated that bone surgery, especially osteotomy, osteosynthesis, ligament repair and reconstruction, treatment of an amputated digit, and longer operative times were risk factors for rescue analgesia use on the first day after upper limb surgery performed under single-injection BPB.

Bone surgery carries a risk of severe postoperative pain because myelinated and unmyelinated afferent nerve fibers, including nociceptors, are present in the bones [7, 8]. Afferents innervating the bone tissue contain neuropeptides commonly associated with nociceptive processing, such as substance P and calcitonin gene-related peptide [9]. Moreover, bone damage can produce higher levels of prostaglandin E2, a pain-enhancing mediator [10], compared to soft tissue damage alone [11, 12]. This indicates that pain after bone surgery can be severe, and these procedures may carry a higher risk of rescue analgesic use compared to soft tissue surgery. The efficacy of NSAIDs for pain relief after orthopedic surgery [13, 14] supports the hypothesis that prostaglandins are critically involved in postoperative bone pain.

Among the bone surgery procedures, osteosynthesis, osteotomy, ligament repair and reconstruction, and treatment for an amputated digit carried the highest risk of rescue analgesic use for postoperative pain. This may be because these procedures all include damage to and involvement of both the bone cortex and medulla. Moreover, nerve fibers are equally abundant in the bone medulla and cortex [15]. In animal experiments, drilling through the tibia and calcaneus resulted in low thresholds to painful stimuli, although periosteum scalping did not change the threshold [8].

A longer duration of surgery was another risk factor for the use of rescue analgesics in the postoperative period. Indeed, lengthy procedures often involve extensive tissue damage, potentially causing an increase in the release of inflammatory mediators [16]. In addition, a tourniquet is often used in limb surgery to obtain a bloodless field-of-view. Although the duration of tourniquet placement was not analyzed in the multi-explanatory-variable logistic regression model in this study because it had collinearity with the duration of surgical procedure, it can be considered a risk factor. In fact, tourniquet-induced ischemia causes tissue damage, the severity of which depends on the duration of ischemia [17]. In support of our results, a previous study reported that postoperative pain is greater with longer applications of a tourniquet and that a long surgical procedure can be considered a risk factor of rescue analgesic use in the postoperative period [18].

Pain intensity and nerve distributions vary between different sites [8, 15]; therefore, we expected to find a relationship between rescue analgesic use and surgical site. However, no such relationship was found in the present study. The mechanism and type of surgical injury may affect postoperative pain to a much greater extent than its location. No sex-related differences in rescue analgesic use were found in our study in multiple-explanatory-variable models. In support of our results, although it was reported that women might have lower pain thresholds, this difference in men is unlikely to have any clinical relevance [5].

There are some limitations to the present study. First, information on the intensity of preoperative acute or chronic pain and the preoperative oral analgesic use in our patients was not collected from medical records. Our patients may have had different thresholds for requesting pain medication, as well as a different history of chronic pain and pre-injury use of pain medications. Second, their preoperative psychiatric states such as anxiety and mood could also have influenced the use of pain medication.

In summary, we determined the risk factors of rescue analgesic use for treating postoperative pain after upper limb surgery performed under single-injection BPB. Based on our findings, we advocate for strengthening analgesia (e.g., using continuous brachial plexus blocks [19] and opioid-based analgesia) in patients with the risk factors identified in this study.

## Abbreviations

BPB: Brachial plexus block
CI: 95% confidence intervals
NSAIDs: Nonsteroidal anti-inflammatory drugs
OR: Odds ratios
